# Moral decision-making in patients with neurodegenerative diseases: a systematic review

**DOI:** 10.3389/fpsyg.2026.1745923

**Published:** 2026-02-12

**Authors:** Giorgia Capitani, Daniele Lozzi, Giuseppe Curcio, Simone Migliore

**Affiliations:** 1 Department of Biotechnological and Applied Clinical Sciences, University of L’Aquila, L’Aquila, Italy; 2 Acquisition, Analysis, Visualization and Imaging Laboratory (A2VI Lab), Department of Life, Health and Environmental Sciences, University of L’Aquila, L’Aquila, Italy

**Keywords:** Alzheimer disease, frontotemporal dementia, moral decision-making, moral dilemma, Parkinson disease, trolley dilemma

## Abstract

**Introduction:**

Moral decision-making, a core component of social cognition, relies on integrating affective and cognitive processes supported by distributed neural networks. Neurodegenerative diseases disrupt these systems to varying degrees, offering unique models to investigate the neural bases of moral cognition. This review aimed to systematically examine moral decision-making deficits across neurodegenerative diseases, delineate disease-specific patterns of moral cognition impairment, and highlight conceptual and methodological gaps to inform future research and clinical assessment.

**Methods:**

A systematic search of PubMed, Web of Science, and Scopus was conducted for studies published up to January 2025, following Preferred Reporting Items for Systematic Reviews and Meta-Analyses 2020 guidelines.

**Results:**

Seventeen studies met inclusion criteria. Convergent evidence indicates that behavioral variant frontotemporal dementia (bvFTD) produces a distinctive utilitarian bias characterized by diminished empathy, emotional blunting, and impaired integration of intention and outcome, reflecting degeneration of the ventromedial prefrontal cortex, anterior insula, and amygdala within the salience and default mode networks. In contrast, Alzheimer’s disease (AD) patients typically preserve affective aversion to harm, suggesting relative sparing of limbic–ventromedial circuits despite conceptual and executive decline. Moral reasoning in Parkinson’s disease (PD) and amyotrophic lateral sclerosis (ALS) remains largely intact unless frontotemporal involvement occurs, while dementia with Lewy bodies (DLB) manifests intermediate profiles marked by reduced cognitive theory of mind and aberrant moral affect.

**Discussion:**

These findings delineate disease-specific patterns of moral dysfunction linked to network-level degeneration rather than global cognitive decline. Understanding these mechanisms holds translational relevance for early diagnosis, ethical capacity assessment, and the development of ecologically valid tools to monitor socio-emotional deterioration in neurodegenerative disorders.

## 1 Introduction

Dementia is an umbrella term for a set of progressive neurodegenerative syndromes characterized by cognitive decline severe enough to interfere with independent daily functioning. Clinically, dementia can result from various underlying diseases that affect the brain, leading to distinct patterns of cognitive and behavioral impairment. In medical and neuropsychological contexts, “dementia” implies not just memory loss but a global cognitive deterioration across domains such as memory, language, executive function, visuospatial skills, and social cognition, often accompanied by changes in behavior and personality (Livingston et al., 2024). However, the exact profile of deficits can differ markedly depending on the type of neurodegenerative disease, as different pathologies target different neural networks and regions of the brain. For example, Alzheimer’s disease (AD) is the most common cause of dementia and is classically characterized by an early and prominent episodic memory impairment, followed by progressive decline in other cognitive domains (Kim et al., 2019). From a neuroanatomical perspective, AD is primarily associated with early degeneration of medial temporal lobe structures, including the hippocampus and entorhinal cortex, followed by progressive involvement of temporoparietal and posterior cingulate regions (Jack et al., 2018). Amnestic Mild Cognitive Impairment (aMCI) represents a prodromal stage of Alzheimer’s disease, characterized by objective episodic memory impairment with relatively preserved daily functioning and partially preserved socio-emotional abilities. Although aMCI does not meet criteria for dementia, it provides a valuable model for studying moral decision-making between healthy aging and AD, helping to disentangle early conceptual and executive contributions to moral reasoning from later affective impairments. In contrast, frontotemporal dementia (FTD) encompasses a group of younger-onset dementia syndromes that primarily affect the frontal and anterior temporal lobes. Clinically, FTD comprises several partially overlapping subtypes with distinct cognitive and behavioral profiles. The behavioral variant frontotemporal dementia (bvFTD) is characterized by early changes in personality, social conduct, and emotional processing, often accompanied by executive dysfunction. In contrast, primary progressive aphasia variants predominantly affect language and communication, with relatively preserved social behavior in the early stages. Despite this clinical heterogeneity, bvFTD is the subtype most consistently associated with socio-emotional and moral decision-making impairments. FTD predominantly affects frontal and anterior temporal cortices, including the ventromedial and orbitofrontal prefrontal cortex, anterior insula, and anterior temporal lobes, with relative sparing of posterior cortical regions in the early stages (Piguet et al., 2011). Parkinson’s disease (PD) is primarily defined by motor symptoms such as bradykinesia, resting tremor, rigidity, and postural instability, reflecting early degeneration of dopaminergic nigrostriatal pathways with secondary disruption of fronto-striatal circuits and later cortical involvement contributing to cognitive and socio-emotional deficits (Balestrino and Schapira, 2020). Parkinson’s disease dementia (PDD) is characterized by deficits that differ from AD. PD patients typically show executive dysfunction, slowed thought processing, attention and working memory deficits, and social cognition deficits, often with relatively milder memory loss in early stages (Rosen et al., 2015). These cognitive changes in PD are attributed to the involvement of fronto-striatal circuits (Williams-Gray et al., 2007; Jellinger, 2023). In addition to the canonical conditions described above, both amyotrophic lateral sclerosis (ALS) and dementia with Lewy bodies (DLB) have been associated with cognitive impairments. Although ALS is primarily a motor neuron disease, frequently shows extra-motor involvement of prefrontal, anterior temporal, and insular regions providing a neuroanatomical basis for executive and socio-emotional deficits when frontotemporal pathology is present. Indeed, a substantial proportion of patients exhibit cognitive and behavioral changes, including executive dysfunction and deficits in social cognition such as theory of mind and emotion recognition, particularly in the context of the ALS–FTD spectrum. These non-motor impairments may influence aspects of moral judgment that depend on cognitive control and socio-emotional integration (Crockford et al., 2018; Palumbo et al., 2022).

DLB involves widespread cortical and subcortical pathology, including posterior cortical regions, limbic structures, and fronto-striatal circuits, driven by combined cholinergic and dopaminergic dysfunction. This pattern underlies attentional fluctuations, visuospatial deficits, and early alterations in social cognition. Such deficits have implications for the ability to process normative social information and complex decision contexts (Kemp et al., 2017).

In summary, Alzheimer’s, frontotemporal, and Parkinson’s disease-related dementias each have distinct neurocognitive profiles: AD predominantly affects memory and cortical thinking abilities, FTD disrupts frontal-executive and socioemotional processes, and PD leads to subcortical-frontal impairments including executive and social cognition deficits. Along this spectrum, ALS—particularly within the ALS–FTD continuum—may involve executive and socio-emotional dysfunction beyond motor impairment, while DLB is characterized by early attentional, executive, and social cognitive deficits linked to widespread fronto-limbic and subcortical pathology. Together, these differences set the stage for understanding how each condition might differentially impact higher-order abilities like moral reasoning and decision-making.

Beyond traditional cognitive domains, increasing attention has been devoted to social cognition, a set of processes that enable individuals to perceive, interpret, and respond appropriately to the social world. Social cognition encompasses multiple partially dissociable abilities, including theory of mind (ToM), emotion recognition, empathy, moral reasoning, and sensitivity to social norms (Arioli et al., 2018). Importantly, ToM does not constitute moral decision-making per se; rather, it represents a foundational social-cognitive capacity that can support moral evaluation by enabling the representation of others’ mental states, without directly determining moral judgments. These functions rely on distributed fronto-temporal, limbic, and insular networks and are particularly vulnerable to neurodegenerative processes that disrupt socio-emotional and self-referential systems (Arioli et al., 2018). Moral cognition represents a core component of social cognition, as it supports judgments and decisions about right and wrong in situations involving others’ welfare, harm, and social norms. Moral decision-making requires the integration of affective signals, contextual information, and goal-directed reasoning, and therefore depends on the coordinated activity of ventromedial prefrontal, limbic, and salience-network regions (Arioli et al., 2018). Neurodegenerative diseases offer a unique model to investigate moral cognition, as selective patterns of network degeneration can differentially preserve or disrupt moral decision-making processes, leading to disease-specific moral profiles (Christidi et al., 2018). Understanding how moral cognition is altered across neurodegenerative conditions is thus crucial for both theoretical models of moral processing and clinical practice. Accumulating evidence suggests that patients with different forms of dementia (i.e., AD, PD, FTD) exhibit changes in moral decision-making, with the most striking deviations reported in FTD.

The present review aimed to map out the literature on moral decision-making deficits in neurodegenerative diseases. It evaluated the evidence for disease-specific patterns of moral cognition impairment and identify key knowledge gaps and methodological challenges in this line of research. The synthesis of findings across major neurocognitive disorders hopes to clarify whether “moral cognition” is differentially affected by distinct neuropathologies and inform future research (and clinical practice) regarding the assessment of moral decision-making in dementia. Ultimately, understanding these changes has not only scientific importance but also ethical and legal implications for the care and social integration of patients with neurodegenerative diseases.

## 2 Materials and methods

The current systematic review was carried out based on the guidelines and principles outlined by the Preferred Reporting Items for Systematic Reviews and Meta-Analyses (PRISMA) statement 2020 and checklist (Page et al., 2021). The review was not preregistered in public registries because it was designed as an exploratory synthesis of a highly heterogeneous and emerging literature on moral decision-making in neurodegenerative diseases, for which paradigms, outcomes, and eligible clinical populations are not yet sufficiently standardized to permit fully prespecified analytic plans.

### 2.1 Search strategy and study selection

A search was conducted on PubMed, Scopus, and Web of Science, refining the results up to January 2025. The same search string was used across all databases, containing the following keywords: “Moral dilemma” OR “moral decision-making” OR “trolley dilemma” OR “moral judgment” AND “neurodegenerative.” The selection of search terms was guided by the conceptual aim of identifying studies explicitly investigating moral decision-making processes in neurodegenerative diseases. Keywords related to moral cognition (e.g., “moral dilemma,” “moral decision-making,” “trolley dilemma,” “moral judgment”) were chosen to capture paradigms involving moral conflict, evaluation of harm, and intention–outcome integration, which constitute the core operationalizations of moral decision-making in the neuropsychological literature. The term “neurodegenerative” was intentionally used as an umbrella keyword to ensure a transdiagnostic search strategy and avoid restricting retrieval to specific disease labels *a priori*. This approach was adopted to maximize sensitivity while allowing disease-specific studies to be identified during the screening phase. Although the search strategy relied on the umbrella term “neurodegenerative,” manual screening of titles and abstracts ensured the inclusion of all disease-specific studies employing moral decision-making paradigms, making it unlikely that relevant conditions were systematically missed. Studies focusing exclusively on cheating or dishonest behavior were excluded, as these paradigms primarily assess moral norm compliance and reward-based decision-making rather than moral reasoning in dilemmatic contexts involving interpersonal harm and moral conflict.

### 2.2 Inclusion and exclusion criteria

The studies included in this review met the following inclusion criteria: they were original research articles, not reviews or systematic reviews, across all three databases considered. For Web of Science, specific categories were excluded, including: Business, Computer Science - Artificial Intelligence, Economics, Ethics, Health Care Science Services, History and Philosophy of Science, Law, Management, Medical Ethics, Nursing, Philosophy, Religion. Additionally, duplicate records were removed using Excel’s ‘Remove Duplicates’ function. Thus, the selected articles primarily focus on moral decision-making in individuals with neurodegenerative diseases, whereas articles with abstracts deemed off topic were excluded. Regarding sample characteristics, studies were included if they involved patients diagnosed with a neurodegenerative disease according to established clinical criteria, regardless of disease stage or severity. The presence of a healthy control group was not mandatory for inclusion, given the exploratory and heterogeneous nature of the available literature. No restrictions were applied based on sample size. With respect to methodology, studies were required to employ tasks explicitly assessing moral decision-making, such as moral dilemmas or moral judgment paradigms involving interpersonal harm, moral conflict, or intention–outcome evaluation. Studies focusing exclusively on related but conceptually distinct constructs (e.g., cheating or reward-based decision-making without moral conflict) were excluded. Neuropsychological, behavioral, physiological, and neuroimaging methodologies were all considered eligible. Only articles written in English and matching the predefined search string were ultimately included. Although articles in languages other than English were not explicitly excluded *a priori*, none met the inclusion criteria in the final screening, as they did not employ explicit moral decision-making paradigms or involved non-empirical or non-clinical samples.

### 2.3 Data extraction and analysis

PRISMA recommendations for systematic literature analysis were strictly followed. Given the heterogeneity of study designs, outcome measures, and experimental paradigms, no formal risk-of-bias tools were applied (e.g., the Newcastle-Ottawa Scale or the Cochrane risk-of-bias instrument), in line with recommendations for systematic reviews of non-interventional and behavioral studies. Instead, study quality was assessed using a structured qualitative framework. Each study was independently evaluated by two reviewers across five predefined domains: (1) Sample characterization and recruitment bias (clarity of diagnostic criteria, inclusion/exclusion criteria, and representativeness of the clinical sample); (2) Assessment validity (use of validated moral decision-making paradigms and appropriate neuropsychological measures); (3) Statistical control (adequacy of analytical methods, control of relevant covariates such as age, education, and global cognitive status); (4) Incomplete outcome data (handling of missing data and attrition); and (5) Reporting transparency (clarity and completeness of methodological and results reporting) (Popay et al., 2006; Higgins et al., 2019). For each domain, studies were rated as strong, moderate, or weak. An overall qualitative judgment was then derived by integrating these domain-level ratings. Studies were classified as strong when no major methodological concerns were identified, moderate when one or more domains showed limitations unlikely to invalidate the findings, and weak when multiple domains showed substantial methodological shortcomings likely to affect interpretability. Discrepancies were resolved through discussion and consensus. In the rare cases where both reviewers rated a study as weak, it was retained for completeness, but its findings were interpreted with caution during synthesis.

## 3 Results

### 3.1 Literature search

Initially, 14,734 articles were selected. First, the duplicates, reviews, systematic reviews, and meta-analyses were removed, resulting in 5955 articles. Then, all the non-relevant articles were excluded and 5,938 studies were selected. Moreover, the studies that did not employ moral decision-making paradigms were also excluded. Overall, 17 studies met the inclusion criteria and were included in the qualitative synthesis. Eleven studies were classified as strong (Torralva et al., 2000; Mendez et al., 2005; Rosen et al., 2013; Baez et al., 2014a, 2014b, 2016a, 2016b, 2023; Van den Stock et al., 2017; Serrano-Pastor et al., 2020; Strikwerda-Brown et al., 2021), reflecting well-defined samples, appropriate control groups, validated tasks, and adequate statistical analyses. Five studies were rated as moderate (Gleichgerrcht et al., 2011; Fong et al., 2017; Antoniou et al., 2023; Baksh et al., 2024; Msika et al., 2024), mainly due to small sample sizes, limited statistical control of potential confounders, or incomplete reporting of some methodological details. Only one study was classified as weak (single-case designs) (Antoniou et al., 2024). bvFTD was the most frequently investigated condition, represented in 12 studies, followed by AD in 7 studies. ALS, primarily within the ALS–FTD spectrum, PD and DLB were each examined in one study. Moral decision-making was most commonly assessed using moral dilemma paradigms, including trolley-type scenarios and moral judgment tasks manipulating intention–outcome integration. Less frequently, moral reasoning or moral emotion tasks were employed. To facilitate interpretation of the heterogeneous moral decision-making paradigms used across studies, a detailed description of each moral test is provided in Supplementary material. Across studies, moral decision-making measures were often combined with assessments of social cognition, executive functions, and, in some cases, neuroimaging or psychophysiological indices. A detailed description of individual study characteristics is provided in Table 1. In organizing Table 1, the participants, cognitive domains assessed, cognitive test, neuroimaging measures, moral decision-making test used, methodological comments and relevant results were considered.

**Table 1:** All studies related to moral decision-making in neurodegenerative disease.

References	Participants (Male/Female, mean years ± SD)	Cognitive domains assessed	Cognitive test	Neuroimaging measures	Moral decision-making test	Methodological comments	Results
1. Baez et al. (2014a, 2014b)	8 PFL patients (6 M/2 F, 47.5 ± 14.3) 19 bvFTD patients (11 M/8 F, 41.4 ± 5.6) 8 HC (for PFL, 6 M/2 F, 46.8 ± 14.7) 19 HC (for bvFTD, 10 M/9 F, 42.4 ± 4.2)	General cognitive state and premorbid IQ, verbal working memory, spatial working memory, abstraction capacity, verbal inhibitory control	MMSE, WAT-BA, IFS	–	Moral judgment task	Between-groups cross-sectional design	Both patients with PFL and those with bvFTD showed abnormal moral judgment stemming from impaired integration of intentions and outcomes. In both groups, attempted harm was judged as more permissible than by HC. bvFTD patients also showed abnormal judgments of accidental harm, being less willing to exonerate those who caused harm unintentionally. bvFTD patients judged accidental harm as less permissible than PFL patients, pointing to subtle differences between groups
2. Baez et al. (2023)	31 bvFTD (14 M/17 F, 71.58 ± 8.97) 30 AD (11 M/19 F, 75.50 ± 6.27) 37 HC (11 M/26 F, 71.73 ± 9.49)	Cognitive and executive functioning, general cognition	MoCA, ACE-III, IFS	MRI	MST	Cross-sectional, observational group comparison study	Patients with bvFTD showed more severe deficits in self-conscious and other-oriented moral emotions compared to AD patients and HC. Assessing moral emotions alongside general cognitive measures helped distinguish bvFTD from AD. In bvFTD, lower moral emotion scores correlated with reduced gray matter volume in the caudate nucleus and temporal lobes, while in AD, they were linked to atrophy in frontal, temporal, and parietal regions
3. Baez et al. (2016a, 2016b)	21 bvFTD (11 M/10 F, 63.80 ± 7.33) 19 HC (9 M/10 F, 60.42 ± 6.77)	General cognitive state, executive functions	MMSE, IFS	MRI	Moral Judgment Task	Observational case–control study	Patients rated attempted harm as more permissible and accidental harm as less permissible than HC. Performance on accidental harm correlated with gray matter volume in the precuneus, and in HC also with the VMPFC. For attempted harm, both groups showed associations with the temporoparietal junction. Poorer performance related to smaller precuneus and temporal pole volumes
4. Baez et al. (2014a, 2014b)	37 bvFTD (22 M/15 F, 66.0 ± 7.43) 30 HC (15 M/15 F, 55.0 ± 8.64)	General cognitive status, social cognition, executive functions	MMSE, IFS, TMT TASIT, RMET	–	SNQ	Cross-sectional case–control study	BvFTD patients presented deficits in affective, cognitive and moral aspects of empathy. However, empathic concern was the only aspect primarily affected in bvFTD that was neither related nor explained by deficits in EF or other social cognition domains. Deficits in the cognitive and moral aspects of empathy seem to depend on EF, emotion recognition and ToM
5. Baez et al. (2016a, 2016b)	26 bvFTD (14 M/12 F, 66.08 ± 7.45) 23 HC (13 M/10 F, 62.69 ± 9.01)	General cognitive state and premorbid IQ, verbal working memory, spatial working memory, abstraction capacity, verbal inhibitory control	MMSE, IFS	MRI	Modified version of moral judgment task	Between-groups cross-sectional design	In bvFTD patients, atrophy of limbic structures (amygdala and anterior paracingulate cortex and APC) was related to impairments in intentionality comprehension, while atrophy of the orbitofrontal cortex (OFC) was associated with empathic concern deficits. Intentionality comprehension impairments were predicted by EFs and orbitofrontal atrophy predicted deficits in empathic concern
6. Baksh et al. (2024)	11 bvFTD – 13 AD – 4 aMCI (18 M/10 F, 62.50 ± 5.96) 28 HC (11 M/17 F, 64.54 ± 7.35)	Executive function, memory, language, visuospatial skills, Verbal fluency	ECAS, BMIPB, TMT, FSCRT, D-KEFS, letter fluency, graded naming test, the Warrington spelling test, TROG, VOSP, ESCoT, RME,	–	SNQ	Cross-sectional case–control study	Patients with aMCI or dementia present marked impairments in affective ToM, overall ESCoT performance, and the RME test compared to HC. Importantly, the ESCoT identified distinct links between social cognitive deficits and behavioral symptoms: Affective ToM correlated with apathy, loss of empathy, perseveration, and psychotic features. Cognitive ToM was associated with disinhibition, loss of empathy, and hyperorality or altered eating behaviors
7. Fong et al. (2017)	10 bvFTD (4 M/7 F, 62.40 ± 11.51) 11 AD (3 M/9 F, 61.36 ± 5.70) 13 HC (4 M/9 F, 53.88 ± 9.51)	Social cognition and executive functions	SCR	–	MBI, SNQ, Moral Dilemmas	Cross-sectional case–control study	bvFTD participants were more willing to harm in the personal, but not the impersonal, dilemma compared to AD and HC groups. BvFTD participants had lower arousal and less of an increase in conflict on the personal versus the impersonal dilemma, in contrast to increased arousal and conflict for the AD and HC groups. Furthermore, bvFTD participants verbalized less discomfort, a correlate of low adherence to social norms
8. Gleichgerrcht et al. (2011)	13 YES group (8 M/5 F, 71.4 ± 5.46) 9 NO group (3 M/6 F, 71.2 ± 6.80)	General cognitive status, verbal memory, nonverbal memory, language, attention, visuospatial abilities, executive functions	MMSE, ACE-R, RAVLT, ROCF, TMT A-B, WCST, IFS, MIE, IGT, IRI	–	MBI	Cross-sectional case–control study	Patients endorsing the utilitarian option (pushing the man) scored significantly lower on affective ToM, particularly on the MIE test, than those who refrained. No group differences emerged in empathy, moral knowledge, executive function, or other cognitive domains, suggesting that altered moral judgment stems from specific affective ToM deficits rather than global cognitive decline
9. Mendez et al. (2005)	26 FTD (12 M/14 F, 60.8 ± 7.6) 26 AD (12 M/14 F, 64.3 ± 9.1) 26 HC (12 M/14 F, 62.3 ± 9.1)	Executive function, memory, language, visuospatial skills, social/moral cognition	MMSE, CDR, CERAD battery, FAB, Boston naming, verbal fluency, memory tasks	–	Moral behavior inventory, trolley/footbridge dilemmas	Cross-sectional, observational group comparison study	All groups (including AD patients and HC) retained knowledge of moral norms and the ability to make impersonal moral judgments. However, FTD patients showed specific deficits in making rapid, emotionally driven moral judgments compared to AD patients and HC
10. Msika et al. (2024)	7 DLB (4 M/3 F, 86.1 ± 4.9) 8 AD (3 M/5 F, 85.1 ± 4.6) 16 HC for DLB (4 M/12 F, 75.1 ± 4.9) 18 HC for AD (9 M/9 F, 80.8 ± 4.6)	General cognitive state, moral cognition	MMSE, ITQ, 10 items related to realism and auditory factors from the presence questionnaire	–	REALSoCog task	A cross-sectional observational case–control study	DLB patients displayed significant deficits in emotional empathy and ToM, along with more inappropriate behavioral responses, especially in conventional social situations. In contrast, AD patients showed relatively preserved social cognition, with only mild cognitive ToM impairments
11. Antoniou et al. (2023)	7 bvFTD (4 M/3 F, 63.1 ± 6.9) 7 HC (4 M/3 F, 73.7 ± 9.9)	General cognitive capacity	MMSE, CDR, GDS	–	Moral reasoning task	Cross-sectional case–control study	bvFTD patients expressed more positive than negative emotions and demonstrated reduced cognitive precision in their moral reasoning compared to HC. Their decisions were primarily guided by kindness, altruism, and rule compliance. Importantly, utilitarian responses in bvFTD did not stem from emotional detachment or antisocial tendencies but were instead driven by positive affect and prosocial motives
12. Antoniou et al. (2024)	1 case study with early bvFTD (1 M, 58)	General functioning, language, visuospatial, memory, executive functioning	MMSE, CDR, GDS, patient competency rating scale (informant), Boston naming task, wide range achievement test, Benson figure—copy, California verbal learning test, digit span, Stroop, TMT, cognitive ToM, IRI, CATS, TASIT-EET, revised self-monitoring scale	MRI	Moral reasoning task, SNQ	Single case study	This study presents the case of Mr. U, a patient with bvFTD who displayed impartial, altruistic utilitarian reasoning despite marked socioemotional deficits, including reduced empathy and emotion recognition. Neuroimaging showed frontal, temporal, and insular atrophy, consistent with impaired emotion and interoceptive processing
13. Rosen et al. (2013)	19 PD (7 M/12 F, 65.16 ± 7.75) 20 HC (7 M/13 F, 62.05 ± 10.02)	General cognitive state, paired learning, Altering verbal fluency, mental rotation, working memory, paired recall, intelligence, executive functions, ToM, decision making	MMSE, PANDA, MWT-B, MCST, RMET, FAS test, GDT, SCR	–	Moral decision-making	Cross-sectional comparative study	The two groups showed no differences in overall moral decision-making performance. Although ToM abilities were similar, ToM was inversely related to altruistic moral choices in HC but not in PD patients. Executive functions were unrelated to moral decisions, and SCR did not differ between groups, though both showed responses above zero
14. Serrano-Pastor et al. (2020)	10 ALSwi (5 M/5 F, 57.7 ± 11.56) 10 ALSbi (6 M/4 F, 58.9 ± 7.52) 10 ALSFTD (4 M/6 F, 62.57 ± 12.56) 23 HC (11 M/12 F, 60.95 ± 7.26)	Executive functions, verbal fluency and language, memory and visuospatial skills	ECAS, FRSBE	–	Moral dilemmas	Cross-sectional case–control study	Impairments in the interpersonal and intrapersonal moral reasoning process were found in ALSFTD patients. Regarding the moral conflict, there are some indications of mild impairment in ALS patients with and without dementia. All these impairments appear to associate with behavioral changes
15. Strikwerda-Brown et al. (2021)	26 bvFTD (21 M/5 F, 62.96 ± 8.27) 14 AD (7 M/7 F, 66.29 ± 7.00) 22 HC (13 M/9 F, 63.32 ± 7.20)	Verbal attention, working memory, language, episodic memory and executive function	ACE-III, ACE-III language, digit span forwards maximum span, digit span backwards maximum span, RAVLT 30-min recall, RCF 3-min recall, trail making test part B-A	MRI	Moral reasoning task	Cross-sectional comparative study	In bvFTD, patients showed normal moral decision patterns compared to AD and HC but reported abnormally positive emotions following high-conflict moral choices, with 61.5% feeling “extremely good” about their decisions. This blunted affective response correlated with reduced social norm knowledge and was linked to atrophy in frontal, subcortical, and lateral temporal regions. Diffusion imaging identified the uncinate fasciculus as the key pathway connecting degraded social conceptual knowledge with impaired emotional reactions during moral conflict
16. Torralva et al. (2000)	25 AD (11 M/14 F, 71.58 ± 8.97) 20 HC (6 M/14 F, 71.73 ± 9.49)	Global cognitive functions, visuospatial reasoning, access to semantic information, executive functions, verbal learning, visual perception and nonverbal memory, auditory attention, constructional praxis	MMSE, Raven’s progressive matrices, Wisconsin card sorting test, controlled oral word association test, Buschke selective reminding test, Benton visual retention test, digit span, block design, similarities, Bechara’s card test	–	Moral judgment interview	Cross-sectional case–control study	AD patients show significant impairments in social judgment and real-life decision-making compared to HC. Poor performance on the Moral Judgment Interview was associated with deficits in visuospatial and abstract reasoning, but not with psychiatric or behavioral symptoms. Similarly, AD patients performed worse on the Bechara Card Test, with results largely explained by verbal and visual memory impairments. Behavioral disturbances, although prevalent in AD, were not significantly related to moral or decision-making deficits
17. Van den Stock et al. (2017)	13 bvFTD (9 M/4 F, 66.6 ± 7.22) 19 HC (11 M/8 F, 66.5 ± 6.28)	Global cognitive ability, verbal memory, categorical verbal fluency, abstract reasoning ability, visual divided attention and task shifting, low- level aspects of visual perception, confrontation naming, language comprehension	MMSE, RAVLT, AVF, RCPMT, TMT, BORB, BNT, AAT	MRI-fMRI	Judgment of moral dilemmas	Cross-sectional observational case–control study	bvFTD patients gave more utilitarian responses in low-conflict personal moral dilemmas. These responses correlated with facial emotion processing scores. Voxel-based morphometry showed that utilitarian choices were linked to reduced gray matter in ventromedial prefrontal regions, and resting-state fractional Amplitude of Low Frequency Fluctuations analyses revealed an additional association with anterior insula activity

AAT, Aachen Aphasia Test; ACE-III, Addenbrooke’s Cognitive Examination-III; ACE-R, Adden brooke’s Cognitive Examination-Revised; AD, Alzheimer disease; ALS, Amiotrophic Lateral Sclerosis; ALSbi, Amiotrophic Lateral Sclerosis behavioral impairment; ALSFTD, Amiotrophic Lateral Sclerosis with frontotemporal dementia; ALSwi, Amiotrophic Lateral Sclerosis patients without cognitive or behavioral impairment; aMCI, amnestic Mild Cognitive Impairment; AVF, Animal Verbal Fluency; BMIPB, BIRT Memory and Information Processing Battery; BNT, Boston Naming Test; BORB, Birmingham Object Recognition Battery; bvFTD, behavioral variant of frontotemporal dementia; CATS, Comprehensive affective testing system; CDR, Clinical Dementia Rating Scale; CERAD battery, Tests from the Consortium to Establish a Registry in Alzheimer’s Disease; D-KEFS, Delis-Kaplan Executive Function System; DLB, patients suffering from Dementia with Lewy Bodies; ECAS, Edinburgh Cognitive and Behavioral Amyotrophic Lateral Sclerosis Screen; EF, Executive Function; ESCoT, Edinburgh Social Cognition Test; FAB, Frontal Assessment Battery; FAS, tests of semantic and letter fluency; FRSBE, Frontal Systems Behavior Scale; FSCRT, Free and selective cued reminding test; FTD, Frontotemoral Temporal Dementia; GDS, Geriatric Depression Scale; GDT, Game of DiceTask; HC, Healthy Control; IFS, INECO frontal screening; IGT, Iowa Gambling Task; IRI, Interpersonal Reactivity Index; ITQ, Immersive Tendencies Questionnaire; MBI, Moral Behavior Inventory; MCST, Modified Card Sorting Test; MIE, Mind in the Eyes Test; MMSE, Mini-Mental State Examination; MoCA, Montreal Cognitive Assessment; MRI, Magnetic Resonance Imaging; MST, Moral Sentiment Task; MWT-B, Mehrfach Wortschatz Test B; NO group, BvFTD patients that answer no to the footbridge moral dilemma; PANDA, Parkinson Neuropsychometric Dementia Assessment; PD, Parkinson Disease; PFL, Prefrontal Lesions; RAVLT, Rey Auditory Verbal Learning Test; RCF, Rey Complex Figure; RCPMT, Raven’s Colored Progressive Matrices; REALSoCog, Task that revealing social cognition deficits; RME, Reading the Mind in the Eyes; RMET, Reading the mind in the eyes test; ROCF, Rey-Osterrich Complex Figure; SCR, skin conductance responses; SNQ, Social Norms Questionnaire; TASIT, The awareness of social inference test; TASIT-EET, The awareness of social interference test abbreviated; TMT, Trail Making Test; ToM, Theory of Mind; TROG, Test for Reception of Grammar; VMPFC, ventromedial prefrontal cortex; VOSP, Visual Object and Space Perception Battery; WAT-BA, word accentuation test; WCST, Wisconsin Card Sorting Test; YES group, BvFTD patients that answer yes to the footbridge moral dilemma.

**Figure 1:**
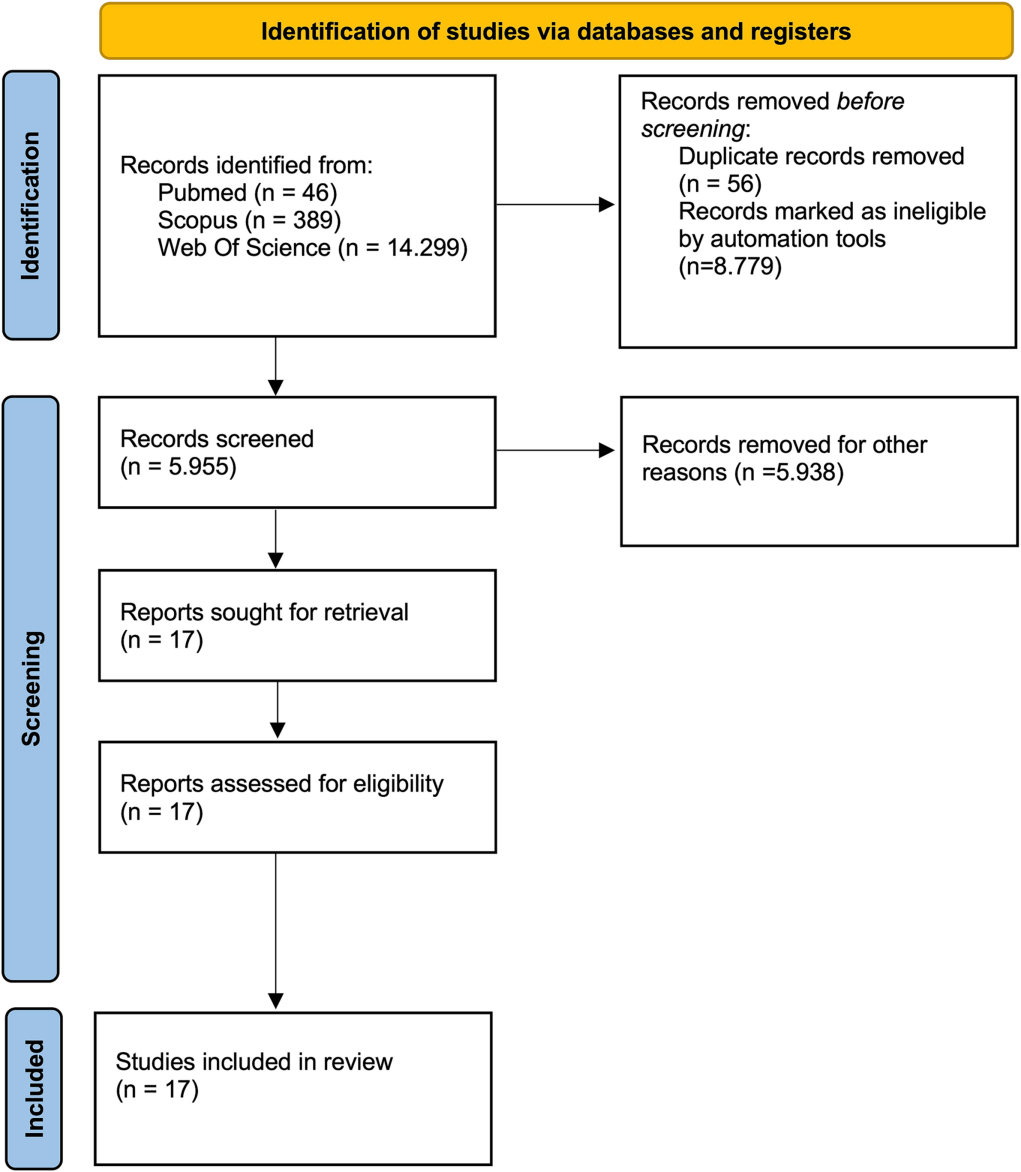
Preferred Reporting Items for Systematic Reviews and Meta-Analyses (PRISMA) diagram of search strategy. Automation tools refer to database-level filters applied prior to screening (e.g., document type and subject category exclusions). “Other reasons” include records excluded during title and abstract screening because they did not involve moral decision-making paradigms, neurodegenerative populations, or original empirical data.

Literature analysis and selection was summarized in a PRISMA-like diagram (see Figure 1).

### 3.2 Moral decision-making in neurodegenerative disease

Across the reviewed studies, moral decision-making was primarily assessed using moral dilemma paradigms (e.g., trolley-type scenarios), moral judgment tasks manipulating intention–outcome integration, and moral reasoning tasks requiring explicit evaluation of morally relevant actions. These paradigms are designed to probe different components of moral cognition, including affective engagement, sensitivity to harm, and the resolution of moral conflict.

Across studies, bvFTD patients consistently show a utilitarian bias in moral decision-making, characterized by reduced emotional engagement, diminished empathy, and impaired integration of intentions and outcomes (Gleichgerrcht et al., 2011; Baez et al., 2014b; Antoniou et al., 2023). These alterations represent a core socio-emotional deficit rather than a general executive dysfunction. Behaviorally, bvFTD patients rely predominantly on outcome-based reasoning, often perceiving accidental harm as intentional and attempted harm as permissible, reflecting impaired affective ToM and blunted sensitivity to guilt or moral conflict (Gleichgerrcht et al., 2011; Baez et al., 2014b, 2016a, 2016b). Moral deficits are especially pronounced in low-conflict scenarios, where attenuated emotional arousal fails to inhibit utilitarian choices (Van den Stock et al., 2017). Neuroimaging evidence reveals that gray matter atrophy in the orbitofrontal cortex, amygdala, and anterior paracingulate cortex correlates with reduced empathic concern and intentionality comprehension, while ventromedial prefrontal and anterior insular dysfunction underlies outcome-driven judgments and weakened affective responses (Baez et al., 2016a, 2016b; Van den Stock et al., 2017). These findings point to an early disruption of the salience and default mode networks, compromising interoceptive and emotional integration required for moral evaluation. In line with this, bvFTD patients frequently report neutral or even positive affect after morally transgressive decisions (Antoniou et al., 2023), indicative of decoupling between salience detection and affective evaluation within the ventromedial prefrontal cortex (vmPFC)-insula-amygdala network. Single-case evidence further supports that impaired interoceptive awareness and emotional intuition may drive utilitarian reasoning, occasionally coexisting with partial insight (Antoniou et al., 2024). Physiological and self-report data converge with imaging results: bvFTD patients exhibit lower autonomic reactivity and reduced emotional conflict during harmful decision-making, consistent with breakdown of vmPFC-insula-amygdala circuitry (Fong et al., 2017). This emotional flattening becomes particularly evident in high-conflict moral dilemmas (e.g., footbridge-type trolley dilemmas involving direct harm to one person to save several others), where degeneration of the anterior temporal lobes and the uncinate fasciculus disrupts the integration of emotional signals with conceptual social knowledge (Strikwerda-Brown et al., 2021). Collectively, these findings delineate a neurobehavioral signature of moral cognition in bvFTD, centering on salience-network degeneration and impaired emotional resonance.

In contrast, Alzheimer’s disease (AD) presents a distinct and comparatively preserved moral-affective profile. While bvFTD patients consistently endorse utilitarian reasoning with reduced guilt and empathy, AD patients typically maintain emotional aversion to direct harm, even in the presence of cognitive decline (Mendez et al., 2005; Fong et al., 2017; Strikwerda-Brown et al., 2021; Baez et al., 2023). Their moral reasoning deficits reflect a loss of social-conceptual judgment rather than emotional indifference. In early AD, the ventromedial and limbic circuits remain relatively intact, supporting continued affective responses and moral restraint (Torralva et al., 2000). Consistent with this pattern, moral emotion recognition is markedly disrupted in bvFTD-particularly for prosocial emotions such as pity, embarrassment, and indignation-while AD patients show relatively preserved moral emotion processing (Baez et al., 2023). This dissociation underscores that bvFTD selectively impairs affective and interoceptive components of moral cognition, whereas AD primarily compromises conceptual reasoning and memory-dependent aspects of social judgment.

Extending beyond the FTD-AD spectrum, other neurodegenerative syndromes reveal variable moral vulnerabilities depending on the neural systems involved. In Parkinson’s disease (PD), moral decision-making remains largely preserved in early stages, with no consistent ToM or autonomic deficits—consistent with relative integrity of limbic-ventromedial networks under dopaminergic modulation (Rosen et al., 2013). Conversely, focal prefrontal lesions (PFL) and bvFTD share impairments in integrating intention and outcome, but only bvFTD exhibits over-attribution of blame for accidental harm, implicating broader fronto-insular-temporal dysfunction (Baez et al., 2014a). In amyotrophic lateral sclerosis (ALS), moral reasoning is generally preserved unless frontotemporal degeneration co-occurs (ALS-FTD), in which case patients display utilitarian and emotionally blunted decision patterns paralleling salience-network impairment (Serrano-Pastor et al., 2020). Broader dementia cohorts (aMCI, AD, bvFTD) demonstrate that affective and cognitive ToM deficits correlate with empathy loss and behavioral dysregulation, particularly affecting interpersonal understanding of social norms, while intrapersonal moral knowledge remains relatively intact (Baksh et al., 2024). Finally, dementia with Lewy bodies (DLB) is associated with impaired recognition of conventional transgressions, reduced cognitive ToM, and inappropriate or positive emotional responses to others’ suffering, reflecting prefrontal-limbic disconnection more pronounced than in AD, where moral judgment typically declines only under high emotional load (Msika et al., 2024).

## 4 Discussion

This systematic review highlights convergent evidence that moral decision-making in neurodegenerative disorders is shaped by the progressive breakdown of distributed neural networks underpinning emotional processing, interoceptive awareness, and social cognition. Across studies, a distinctive moral-affective phenotype emerges in bvFTD, characterized by utilitarian reasoning, emotional blunting, and impaired integration of intention and outcome information (Gleichgerrcht et al., 2011; Baez et al., 2014b; Antoniou et al., 2023). These abnormalities reflect a primary socio-emotional deficit rather than a secondary effect of executive dysfunction, emphasizing that moral cognition depends critically on the integrity of limbic-frontoinsular circuits.

Neuroimaging findings consistently implicate degeneration of the orbitofrontal cortex (OFC), amygdala, anterior paracingulate cortex (aPCC), and vmPFC in bvFTD (Baez et al., 2016a, 2016b; Van den Stock et al., 2017). These regions belong to the salience network (SN) and default mode network (DMN)—systems that coordinate affective appraisal and perspective-taking. Damage to these hubs disrupts the dynamic interplay between internal emotional states and external social cues, leading to diminished empathic concern and outcome-based moral judgments (Baez et al., 2016b). The association between OFC and aPCC atrophy and deficits in intentionality comprehension suggests that bvFTD patients lose the capacity to simulate others’ mental states at an emotional level, relying instead on concrete behavioral outcomes. This mechanistic dissociation aligns with models of moral cognition that distinguish between affective valuation (vmPFC-amygdala-insula) and cognitive reasoning (dorsolateral prefrontal and temporoparietal regions).

Physiological and self-report data converge with these structural findings. The markedly lower autonomic reactivity and positive emotional valence following moral transgressions observed in bvFTD (Fong et al., 2017; Antoniou et al., 2023) indicate a decoupling between affective arousal and moral conflict. In healthy individuals, autonomic activation during moral evaluation is mediated by the vmPFC-insula-amygdala circuit, which signals violations of social norms through visceral feedback. In bvFTD, degeneration of these regions likely attenuates this interoceptive signal, impairing the emotional “brake” that normally restrains utilitarian choices (Strikwerda-Brown et al., 2021). Moreover, the involvement of the anterior temporal lobes (ATLs) and uncinate fasciculus (Strikwerda-Brown et al., 2021) further disrupts the integration of conceptual social knowledge with affective cues, resulting in moral reasoning detached from emotional and contextual nuances.

By contrast, AD exhibits a relatively preserved moral-affective profile despite cognitive deterioration. Although AD patients show reduced abstraction and impaired social-conceptual reasoning (Torralva et al., 2000), they typically maintain emotional aversion to harming others (Mendez et al., 2005; Fong et al., 2017; Baez et al., 2023). This dissociation highlights the early sparing of limbic and vmPFC regions, which sustain emotional empathy even as executive and memory processes decline. Thus, moral judgment in AD may depend more on preserved affective intuitions than on higher-order reasoning, contrasting sharply with the affective desensitization seen in bvFTD. The selective vulnerability of SN and DMN in bvFTD versus medial temporal and parietal regions in AD delineates distinct neurocognitive routes to moral impairment - emotional disengagement in the former, conceptual degradation in the latter.

Other neurodegenerative syndromes display variable moral disruption depending on which neural circuits are affected. PD patients generally preserve moral reasoning, consistent with the relative integrity of limbic-ventromedial networks under dopaminergic modulation (Rosen et al., 2013). The relative preservation of moral decision-making observed in Parkinson’s disease should not be interpreted as an absence of vulnerability in the underlying neural systems. Growing evidence indicates that non-motor cortico–striato–limbic circuits, modulated by dopamine, play a crucial role in value-based decision-making, uncertainty processing, and executive control. Dopaminergic dysfunction in PD affects these circuits in a heterogeneous manner, depending on disease progression and medication status, leading to alterations in reward sensitivity, risk evaluation, and cognitive flexibility rather than frank moral impairment (Cools et al., 2022; Colautti et al., 2023). From this perspective, moral decision-making in PD may remain behaviorally intact while relying on compensatory mechanisms within limbic–ventromedial networks. Importantly, subtle changes in affective valuation and conflict monitoring may still emerge under conditions of high ambiguity or emotional load, supporting the view that moral cognition in PD reflects a dynamic balance between dopaminergic modulation and network-level resilience rather than a categorical preservation or impairment (Ponsi et al., 2021).

ALS and DLB further support the notion that moral decision-making deficits emerge selectively when fronto-limbic and socio-emotional networks are compromised. In ALS, moral reasoning is generally preserved in patients without cognitive or behavioral involvement; however, when ALS falls within the ALS–FTD spectrum, patients exhibit impairments in resolving moral conflict, increased utilitarian tendencies, and reduced emotional engagement, mirroring the salience-network disruption observed in bvFTD (Serrano-Pastor et al., 2020). These findings suggest that moral alterations in ALS are not intrinsic to motor degeneration but reflect the extension of pathology to frontotemporal and limbic circuits. In DLB, emerging evidence indicates a distinct profile characterized by impaired cognitive theory of mind, reduced sensitivity to social conventions, and inappropriate affective responses to others’ distress. Compared to Alzheimer’s disease, DLB patients show greater disruption of prefrontal–limbic connectivity, likely driven by combined dopaminergic and cholinergic dysfunction, which may undermine the integration of contextual information and emotional appraisal during moral evaluation (Msika et al., 2024). Although the literature remains limited, these findings position DLB as an intermediate phenotype between AD and bvFTD, marked by partial preservation of moral norms but altered socio-emotional modulation. Collectively, these syndromes underscore that moral cognition deteriorates not uniformly but selectively, depending on whether degeneration affects networks mediating emotion-reason integration (vmPFC-insula-amygdala) or social-conceptual knowledge (ATLs-TPJ).

**Figure 2:**
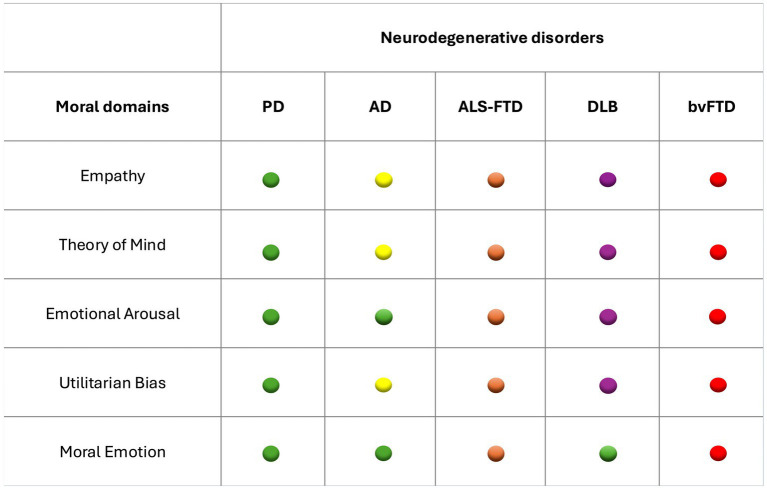
Moral cognition deficits follow a gradient from preserved affective engagement in PD to severe utilitarian and emotionally blunted reasoning in bvFTD. PD, Parkinson Disease; AD, Alzheimer Disease; ALS-FTD, amyotrophic lateral sclerosis-frontotemporal dementia; DLB, dementia with Lewy bodies; bvFTD, behavioral variant frontotemporal dementia. Green: Preserved; Yellow: mild impairment; Orange: moderate impairment; Purple: high fronto-limbic deficit; Red: severe affective–moral disengagement.

**Figure 3:**
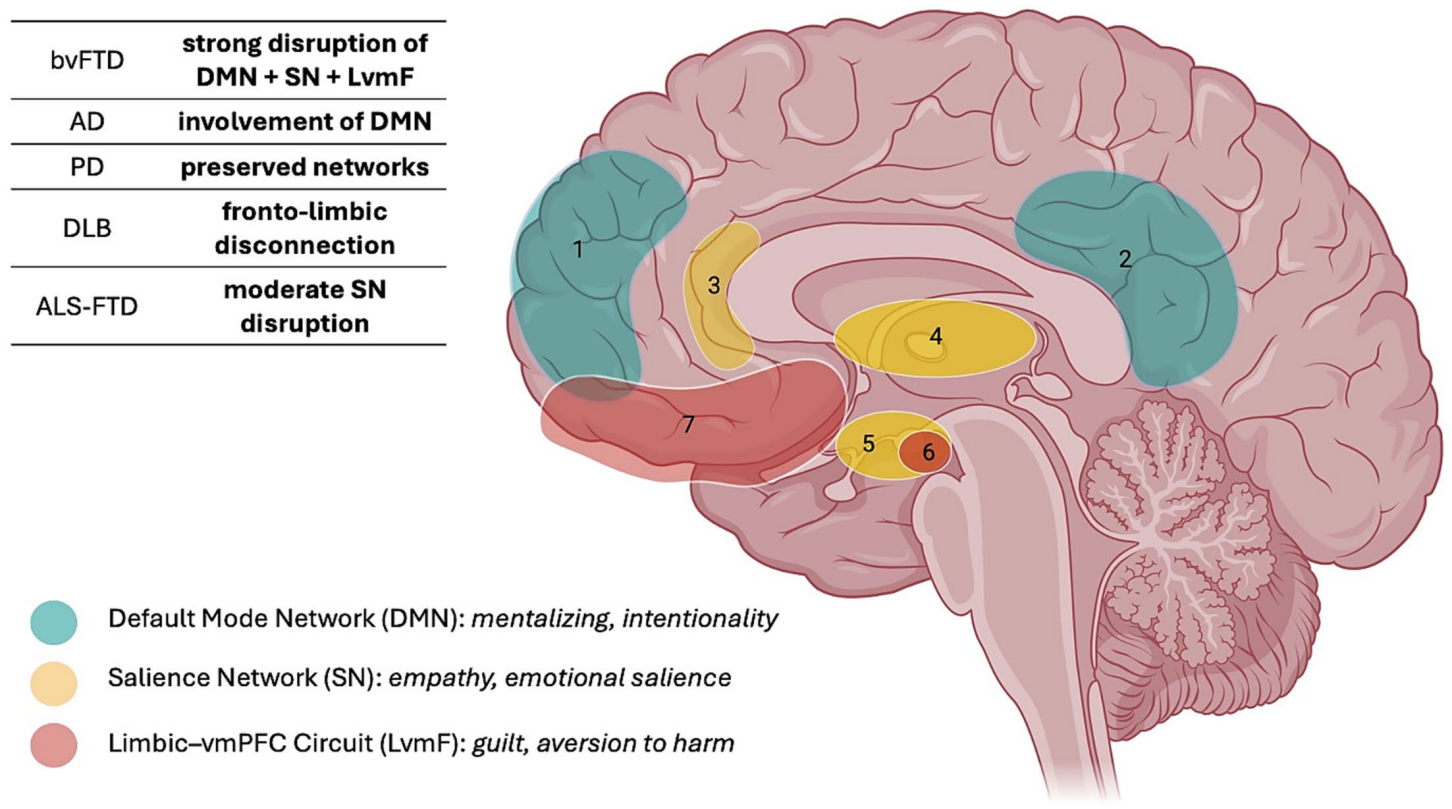
Moral cognition network disruption across neurodegenerative disorders. BvFTD, behavioral variant frontotemporal dementia; AD, Alzheimer’s disease; PD, Parkinson’s disease; DLB, dementia with Lewy bodies; ALS-FTD, amyotrophic lateral sclerosis-frontotemporal dementia. 1: Medial prefrontal cortex; 2: posterior cingulate; 3: anterior cingulate; 4: thalamus + ventral striatum; 5: hypothalamus + amygdala; 6: amygdala; 7: ventromedial prefrontal cortex + orbitofrontal cortex. The figure was created with BioRender.com.

Figure 2 summarizes, across the different neurodegenerative conditions examined, the specific domains of moral cognition that are impaired, whereas Figure 3 illustrates the main neural circuits underpinning these alterations. Together, these visual models provide an integrated overview of the behavioral and neuroanatomical dimensions of moral dysfunction in neurodegenerative diseases.

From a neuropsychological perspective, these findings reinforce a network-based model of moral cognition, wherein ethical decision-making emerges from the interaction between emotional valuation, mentalizing, and executive regulation. The evidence positions bvFTD as a model disorder for studying how affective and interoceptive deficits reshape moral behavior. The observed pattern—preserved rule knowledge but impaired emotional resonance—supports theoretical frameworks such as the Dual Process Theory of moral cognition (Greene et al., 2004), which posits that moral decisions arise from the competition between fast, emotion-driven processes and slower, deliberative reasoning. In bvFTD, the emotional component collapses, leaving a coldly rational, utilitarian bias that lacks the modulatory influence of empathy and guilt.

From a functional neuroanatomical perspective, the presence or absence of moral decision-making deficits across neurodegenerative diseases can be interpreted in relation to the extent and topography of network degeneration. Conditions such as bvFTD, which prominently affect the ventromedial prefrontal cortex, anterior insula, amygdala, and anterior temporal regions, consistently show impairments in integrating emotional signals with intention–outcome evaluation, leading to utilitarian and emotionally blunted moral judgments. In contrast, disorders in which these limbic–ventromedial circuits are relatively preserved—such as early Alzheimer’s disease or non-demented Parkinson’s disease—tend to maintain affective aversion to harm despite deficits in executive or conceptual processing. Moral decision-making appears particularly vulnerable when degeneration disrupts hubs supporting emotion–reason integration, including the salience network and its interaction with default mode regions involved in mentalizing and self-referential processing. Conversely, damage primarily affecting dorsolateral prefrontal or posterior cortical regions may impair deliberative reasoning or contextual integration without necessarily abolishing moral emotions. This functional dissociation helps explain why moral deficits are not uniformly present across neurodegenerative conditions and underscores that moral cognition depends less on global cognitive decline than on the selective involvement of specific socio-emotional networks. See Figure 3 for more details regarding neurodegenerative disorders and neural networks involved in moral decision-making processes.

From a clinical perspective, these findings have relevant practical implications. Traditional executive tests alone may fail to capture socio-emotional decision deficits in bvFTD. Integrating ecologically valid tasks (e.g., moral dilemma paradigms, empathy and emotion recognition measures) with structural and functional imaging could improve diagnostic specificity and early detection. Moral decision-making tasks capture socio-emotional dysfunctions that are often insufficiently assessed by standard neuropsychological batteries, particularly in disorders such as bvFTD and ALS–FTD. Incorporating moral dilemma paradigms, alongside measures of empathy and theory of mind, may improve early differential diagnosis and help identify patients at risk for socially inappropriate or ethically compromised behaviors. Moreover, neurophysiological indices - such as autonomic reactivity - might serve as biomarkers for tracking the progression of social and moral dysfunction (Fong et al., 2017; Strikwerda-Brown et al., 2021; Antoniou et al., 2023). Again, understanding disease-specific moral profiles has implications for evaluating decision-making capacity, legal responsibility, and informed consent, especially in conditions where moral emotions and interoceptive signals are selectively disrupted. Finally, these insights may inform caregiver education and tailored interventions aimed at managing social and ethical challenges in everyday clinical practice.

The present review adds value to the existing literature in several ways. This work provides a systematic synthesis of moral decision-making across multiple neurodegenerative diseases. The review integrates behavioral, physiological, and neuroimaging evidence to show that moral dysfunction reflects selective vulnerability of socio-emotional circuits rather than global cognitive decline. This approach allows for the identification of convergent and divergent moral profiles across diseases, highlighting bvFTD as a prototypical model of affective moral disruption while situating AD, PD, ALS, and DLB along a continuum of network involvement. As such, the present review offers a unifying conceptual framework that can guide future empirical studies and the development of ecologically valid assessment tools (Fong et al., 2017; Strikwerda-Brown et al., 2021; Antoniou et al., 2023)

### 4.1 Limitations

Interpretation of the reviewed evidence is constrained by several methodological limitations. First, moral decision-making paradigms varied substantially across studies, including differences in emotional salience (e.g., personal vs. impersonal dilemmas), ecological validity (hypothetical vignettes vs. more naturalistic scenarios), and response formats (binary permissibility judgments, forced-choice decisions, or ratings). This heterogeneity limits direct comparability across conditions and may partially explain divergent findings, as tasks with higher emotional load may be more sensitive to fronto-limbic dysfunction, whereas low-salience or abstract formats may preferentially capture deliberative or conceptual components of moral evaluation. Second, sample sizes were highly uneven (and often small), especially in rarer clinical groups (e.g., ALS–FTD and DLB), reducing statistical power and increasing susceptibility to sampling bias and inflated effect variability; consequently, null findings in smaller cohorts should be interpreted cautiously. Third, disease severity and clinical heterogeneity were not consistently assessed or controlled across studies. Where severity indices were reported (e.g., global cognitive screening, dementia staging, or disease-specific functional measures), they were not uniformly incorporated into analyses, and important moderators (e.g., medication status in PD, behavioral symptom burden, or cognitive/behavioral status in ALS-spectrum disorders) were variably documented. This limits causal inference regarding whether moral decision-making alterations reflect disease-specific network vulnerability versus broader effects of progression or comorbid neuropsychiatric features. Future research would benefit from (i) greater standardization of moral task batteries (including explicit manipulation of emotional salience and intention–outcome structure), (ii) adequately powered designs (ideally multi-site) with balanced group sizes, and (iii) systematic characterization and statistical control of disease stage/severity and key clinical modifiers to clarify when and why moral cognition diverges across neurodegenerative syndromes.

## 5 Conclusion

Moral cognition provides a sensitive window into the breakdown of socio-emotional networks in neurodegenerative disease. Across disorders, bvFTD emerges as the prototypical model of network-level moral impairment, where degeneration of the vmPFC, anterior insula, and amygdala leads to diminished empathy, emotional indifference, and outcome-based moral reasoning. In contrast, AD and other conditions preserve moral emotions longer, underscoring the differential vulnerability of affective versus conceptual systems. The integration of neuropsychological, physiological, and neuroimaging evidence highlights that moral dysfunction in neurodegeneration reflects not only cognitive decline but a fundamental disconnection between emotion and reason. Future work combining longitudinal imaging with ecologically valid social tasks may elucidate early biomarkers of moral change and inform targeted interventions to preserve social and ethical functioning in affected individuals.
