# Supplementary material for: Moral decision-making in patients with neurodegenerative diseases: a systematic review
Source: Front Psychol. 2026 Feb 12;17:1745923. doi: 10.3389/fpsyg.2026.1745923 (PMC12935944; doi:10.3389/fpsyg.2026.1745923)
Supplement: Supplementary file 1 [file Table_1.docx]

**Moral Judgement Task:** The task comprises 24 moral scenarios, each presented in four versions according to a 2 × 2 factorial design manipulating intention (harmful vs. neutral) and outcome (harmful vs. neutral). In each scenario, protagonists either believe they will cause harm or not, and either actually cause harm or not, such that each belief is true for one outcome and false for the other. This yields four conditions: no harm, accidental harm, attempted harm, and successful harm. After reading each scenario, participants rate how morally permissible the action is on a 7-point Likert scale ranging from totally forbidden (1) to totally permissible (7).

**Moral sentiment task**: The task consists of 29 scenarios describing social situations in which participants perform or observe others’ behaviors. Each scenario is written in the second person and includes two short sentences, one describing the situation and the other the outcome. After reading each scenario, participants select, from a forced-choice list, the emotional category they would feel in that situation, with four response options (one correct, two incorrect, and one neutral) presented in random order. The target emotional categories include guilt, pity, embarrassment, indignation, fear, disgust, and neutrality. In addition, participants rate their emotional experience by indicating the intensity of the emotion and the degree to which it affects them, using 10-point visual analog Likert scales ranging from 0 (low intensity/concern) to 10 (high intensity/concern).

**Social Norms Questionnaire:** This questionnaire consists of 22 yes–no questions. Participants determine whether a behavior is appropriate in the presence of an acquaintance (not a close friend or family member) according to mainstream culture.

**Moral Behavior Inventory:** This questionnaire is a self-report instrument designed to assess moral values, judgments, and behavioral tendencies. It consists of 24 items presenting moral scenarios or statements, and respondents indicate their judgments or likelihood of engaging in those behaviors using 7-point visual analog Likert scales.

**Moral Dilemma**: Participants complete moral dilemma tasks involving either impersonal harm (trolley “switch”) or personal harm (trolley “footbridge”). In the impersonal condition, participants decide whether to divert a trolley using a switch to kill one person instead of five; in the personal condition, they decide whether to push a person onto the tracks to stop the trolley. After each dilemma, participants indicate whether they would perform the action (yes/no), with response times recorded as an index of moral conflict, and then describe how they feel about their decision, which is coded for emotional valence on a 3-point scale (absent, somewhat present, clearly present).

**Trolley/Footbridge Dilemma**: Participants are presented with two trolley-type moral dilemmas read aloud as vignettes: the standard “switch” dilemma and the “footbridge” dilemma. In the switch dilemma, participants decide whether to divert a runaway trolley to kill one person instead of five; in the footbridge dilemma, they decide whether to push a stranger onto the tracks to stop the trolley and save five people. After each vignette, participants restate the scenario and then indicate their decision with a forced-choice yes/no response.

**REALSoCog task:** Participants navigate in a non-immersive virtual city from a first-person perspective along a predefined path and encounter 27 social situations. Sixteen experimental situations are designed to assess social cognition, including moral and conventional norm transgressions, empathic reactions to others in distress, and respect for prohibitions; 11 additional situations serve as normative, emotionally neutral, or positive controls.

For each situation, participants judge whether it is appropriate or inappropriate, rate the severity of any transgression on a 5-point scale, and explain what they have understood. They also indicate whether they would react and, if so, describe their intended behavior, which is coded for appropriateness. For experimental situations, additional questions assess theory of mind and emotional empathy. Participants judge whether a transgressive behavior is intentional and rate its valence (malicious to benevolent), identify how the affected person feels and how intensely, and report their own emotional reaction in terms of intensity and valence.

**Moral Reasoning Task**: Participants complete a semi-structured interview including a moral reasoning task with seven dilemmas assessing utilitarian decision-making. The dilemmas cover three moral categories reflecting positive and negative dimensions of utilitarianism: special obligations (three items evaluating preferences for close others versus the greater good), agent-centered permissions (two items assessing willingness to sacrifice personal interests to benefit others), and personal rights (two items assessing willingness to violate an individual’s rights to maximize overall welfare, e.g., trolley-type dilemmas). Responses are recorded on a 4-point Likert scale (definitely/probably yes/no), yielding a total utilitarianism score (max = 28), as well as separate scores for special obligations (max = 12), agent-centered permissions (max = 8), and personal rights (max = 8), with lower scores indicating more utilitarian reasoning.

**Moral Decision-Making Task**: Participants are presented with 20 short stories on a computer screen describing realistic everyday moral dilemmas. Each story is followed by a forced-choice yes/no question asking whether the participant would choose a proposed behavior in that situation. The dilemmas are designed to be highly imaginable and personally involving and are divided into high-emotional and low-emotional subsets. Across all conditions, half of the proposed behaviors are altruistic and half are egoistic, counterbalanced between emotional categories. Participants give their responses verbally, which are recorded by the experimenter.

**Moral Judgement Interview**: This semi-standardized interview assesses the developmental level of moral reasoning used to resolve hypothetical moral dilemmas. It consists of three parallel forms, each containing three dilemmas, with each dilemma followed by 9–12 standardized probe questions designed to elicit justifications, elaborations, and clarifications of the participant’s moral judgments.

**Judgment of moral dilemmas**: Participants are presented with 50 verbally described hypothetical scenarios, each ending with a question asking whether they would perform a specified action. The scenarios have a uniform grammatical structure and are classified into three categories: non-moral dilemmas (practical decisions; n = 18), impersonal moral dilemmas (weighing harms and benefits impersonally; n = 11), and personal moral dilemmas (utilitarian violations of personal rights; n = 21). Personal moral dilemmas are further divided into high-conflict (n = 13) and low-conflict (n = 8) conditions based on the level of emotional conflict between utilitarian benefit and emotional aversion. Participants respond yes or no to each scenario.
